# Transforming growth factor β signaling in uterine development and function

**DOI:** 10.1186/2049-1891-5-52

**Published:** 2014-11-14

**Authors:** Qinglei Li

**Affiliations:** Department of Veterinary Integrative Biosciences, College of Veterinary Medicine and Biomedical Sciences, Texas A&M University, College Station, TX 77843 USA

**Keywords:** Decidualization, Development, Embryonic development, Implantation, Myometrium, Pregnancy, Transforming growth factor β, Uterus

## Abstract

Transforming growth factor β (TGFβ) superfamily is evolutionarily conserved and plays fundamental roles in cell growth and differentiation. Mounting evidence supports its important role in female reproduction and development. TGFBs1-3 are founding members of this growth factor family, however, the *in vivo* function of TGFβ signaling in the uterus remains poorly defined. By drawing on mouse and human studies as a main source, this review focuses on the recent progress on understanding TGFβ signaling in the uterus. The review also considers the involvement of dysregulated TGFβ signaling in pathological conditions that cause pregnancy loss and fertility problems in women.

## Introduction

Transforming growth factor β (TGFβ) superfamily proteins are versatile and fundamental regulators in metazoans. The TGFβ signal transduction pathway has been extensively studied. The application of mouse genetic approaches has catalyzed the identification of the roles of core signaling components of TGFβ superfamily members in reproductive processes. Recent studies using tissue/cell-specific knockout approaches represent a milestone towards understanding the *in vivo* function of TGFβ superfamily signaling in reproduction and development. These studies have yielded new insights into this growth factor superfamily in uterine development, function, and diseases. This review will focus on TGFβ signaling in the uterus, primarily using results from studies with mice and humans.

### TGFβ superfamily

#### Core components of the TGFβ signaling pathway

Core components of the TGFβ signaling pathway consist of ligands, receptors, and SMA and MAD (mother against decapentaplegic)-related proteins (SMAD). TGFβ ligands bind to their receptors and impinge on SMADs to activate gene transcription. TGFβ superfamily ligands include TGFβs, activins, inhibins, bone morphogenetic proteins (BMPs), growth differentiation factors (GDFs), anti-Müllerian hormone (AMH), and nodal growth differentiation factor (NODAL). Seven type I (i.e., ACVRL1, ACVR1, BMPR1A, ACVR1B, TGFBR1, BMPR1B, and ACVR1C) and five type II receptors (i.e., TGFBR2, ACVR2, ACVR2B, BMPR2, and AMHR2) have been identified [1–4]. SMADs are intracellular transducers. In mammalian species, eight SMAD proteins have been identified and are classified into receptor-regulated SMADs (R-SMADs; SMAD1, 2, 3, 5, and 8), common SMAD (Co-SMAD), and inhibitory SMADs (I-SMADs; SMAD6 and SMAD7). R-SMADs are tethered by SMAD anchor for receptor activation (SARA) [5]. In general, SMAD1/5/8 mediate BMP signaling, whereas SMAD2/3 mediate TGFβ and activin signaling. SMAD6 and SMAD7 can bind type I receptors and inhibit TGFβ and/or BMP signaling [6, 7]. A plethora of ligands versus a fixed number of receptors and SMADs suggests the usage of shared receptor(s) and SMAD cell signaling molecules in this system.

#### TGFβ signaling paradigm: canonical versus non-canonical pathway

To initiate signal transduction, a ligand forms a heteromeric type II and type I receptor complex, where the constitutively active type II receptor phosphorylates type I receptor at the glycine and serine (GS) domain. Subsequent phosphorylation of R-SMADs by the type I receptor and formation and translocation of R-SMAD-SMAD4 complex to the nucleus are critical steps for gene regulation [2, 8–10]. Activation of transcription is achieved by SMAD binding to the consensus DNA binding sequence (AGAC) termed SMAD binding element (SBE) [11, 12], in concert with co-activators and co-repressors. Of note, SMADs can promote chromatin remodeling and histone modification, which facilitates gene transcription by recruiting co-regulators to the promoters of genes of preference [13].

**Figure 1:**
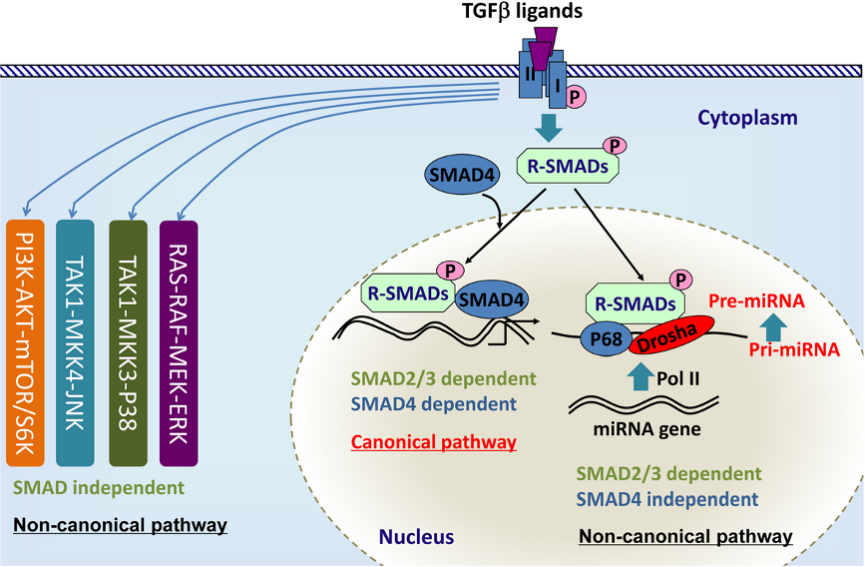
**Canonical and non-canonical TGFβ signaling.** In the canonical pathway, TGFβ ligands bind to serine/threonine kinase type II and type I receptors and phosphorylate R-SMADs, which form heteromeric complexes with SMAD4 and translocate into the nucleus to regulate gene transcription. The non-canonical pathway generally refers to the SMAD-independent pathway such as PI3K-AKT, ERK1/2, p38, and JNK pathways. Recent studies have identified an “R-SMAD-dependent but SMAD4-independent” non-canonical pathway that regulates miRNA maturation.

TGFβ signals through both SMAD-dependent (i.e., canonical) and SMAD-independent (i.e., non-canonical) pathways in a contextually dependent manner [2, 8, 14–16] (Figure 1). The non-canonical pathways serve to integrate signaling from other signaling cascades, resulting in a quantitative output in a given context. Davis and colleagues [17] have recently suggested the presence of microRNA (miRNA)-mediated non-canonical pathway, where TGFβ signaling promotes the biosynthesis of a subset of miRNAs via interactions between R-SMADs and a consensus RNA sequence of miRNAs within the DROSHA (drosha, ribonuclease type III) complex [17–19]. Thus, this type of non-canonical signaling requires R-SMADs but not SMAD4. Multiple regulatory layers including ligand traps (e.g., follistatin), inhibitory SMADs, and interactive pathways exist to determine the signaling output and precisely control TGFβ signaling activity [4, 8, 20–23]. For instance, the linker region of R-SMADs is subject to the phosphorylation modification by mitogen-activated protein kinases (MAPKs) [24]. Therefore, the variable responses triggered by this growth factor superfamily and the complex signaling circuitries within a given cell population underscore the importance of a fine-tuned TGFβ signaling system at both the cellular and systemic levels.

#### TGFβ superfamily signaling regulates female reproduction

**Figure 2:**
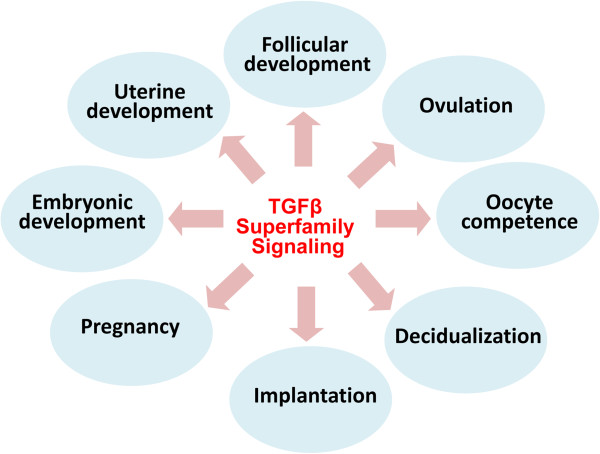
**Major functions of TGFβ superfamily signaling in the female reproduction.** TGFβ superfamily signaling regulates a variety of reproductive processes including follicular development (e.g., TGFβs, GDF9, BMP15, activins, and AMH), ovulation (e.g., GDF9), oocyte competence (e.g., GDF9 and BMP15), decidualization (e.g., BMP2 and NODAL), implantation (e.g., ALK2-mediated signaling), pregnancy (e.g., BMPR2-mediated signaling), embryonic development (e.g., TGFβs, activins, follistatin, BMP2, and BMP4), and uterine development (TGFBR1-mediated signaling).

TGFβ superfamily is evolutionarily conserved and plays fundamental roles in cell growth and differentiation. The signal transduction and biological functions of this signaling pathway have been extensively investigated [2, 4, 8, 9, 25]. TGFβ superfamily signaling is essential for female reproduction (Figure 2), and dysregulation of TGFβ signaling may cause catastrophic consequences, leading to reproductive diseases and cancers [26–33].

Recent studies have uncovered the roles of key receptors and intracellular SMADs of this pathway in female reproduction. *Smad1* and *Smad5* null mice are embryonically lethal, but *Smad8* null mice are viable and fertile [34, 35]. SMAD1/5 and ALK3/6 act as tumor suppressors with functional redundancy in the ovary [27, 29]. *Smad3*^Δex8^ mice demonstrate impaired follicular growth and atresia, altered ovarian cell differentiation, and defective granulosa cell response to follicle-stimulating hormone (FSH) [36, 37]. We have shown that SMAD2 and SMAD3 are redundantly required to maintain normal fertility and ovarian function [38]. Disruption of *Smad4* signaling in ovarian granulosa cells leads to premature luteinization [39]. However, oocyte-specific knockout of *Smad4* causes minimal fertility defects in mice [40]. SMAD7 mediates TGFβ-induced apoptosis [41] and antagonizes key TGFβ signaling in ovarian granulosa cells [42], suggesting inhibitory SMADs are potentially novel regulators of ovarian function. Recent studies show that TGFBR1 is indispensable for female reproductive tract development [43, 44], while ALK2 and BMPR2 are required for uterine decidualization and/or pregnancy maintenance [45, 46].

### TGFβ signaling in uterine development

The uterus develops from the Müllerian duct, which forms at embryonic day E11.75 in mice [47]. Uterine mesenchymal cells remain randomly oriented and undifferentiated until after birth. Between birth and postnatal day 3, circular and longitudinal myometrial layers are differentiated from the mesenchyme [48]. The uterus acquires basic layers and structures by postnatal day 15 [48, 49]. Maturation of the myometrium continues into adulthood. Mechanisms controlling myometrial development are poorly defined. Wingless-type MMTV integration site family (Wnt)*7a* null females demonstrate defects in reproductive tract formation, suggesting a critical role of Wnt/β catenin signaling in myometrial development [50–53].

Myometrial contractility is critical for successful pregnancy and labor. The myometrial cells transform from a quiescent to a contractile phenotype trigged by the decline of progesterone levels during late pregnancy. What has long puzzled scientists is how this transformation occurs during pregnancy, and how myometrial development and function are coordinately regulated. Uterine contraction is controlled by hormonal, cellular, and molecular signals [54–65]. Recent studies have discovered that miRNAs are key regulators of contraction-associated genes and suppressors including oxytocin receptor (*Oxtr*), cyclooxygenase 2 (*Cox2*), connexin 43 (*Cx43*), zinc finger E-box binding homeobox 1 (*Zeb1*), and *Zeb2* [65, 66]. However, signaling pathways that control the development of morphologically normal and functionally competent myometrium are poorly understood.

TGFβ signaling plays a pleiotropic role in fundamental cellular and developmental events [2, 3, 8]. Using a *Tgfbr1* conditional knockout (cKO) mouse model created using anti-Müllerian hormone receptor type 2 (*Amhr2*)-Cre, we have shown that TGFβ signaling is essential for smooth muscle development in the female reproductive tract [43, 44]. The female mice develop a striking oviductal phenotype that includes a diverticulum. The *Tgfbr1* cKO mice are infertile and embryos are unable to be transported to the uterus due to the presence of the physical barrier of oviductal diverticula [43]. Meanwhile, disrupted uterine smooth muscle formation is another prominent feature in these mice, which is associated with a developmental failure of the myometrium during early postnatal uterine development [44]. However, the expression of the majority of smooth muscle genes in the uterus of the conditional knockout mice does not significantly differ from that of controls, suggesting that the developmental abnormality might not be a direct result of intrinsic deficiency in smooth muscle cell differentiation. Our studies point to the contributions of reduced deposition of extracellular matrix proteins, derailed signaling of platelet-derived growth factors, and potentially altered migration of uterine cells during a critical time window of development [44]. The *Tgfbr1* cKO mouse model can be further exploited to understand the pathogenesis of myometrium-associated diseases, such as adenomyosis that is present in these mice [44].

### TGFβ signaling and uterine function

Pre-implantation embryonic development refers to a period from fertilization to blastocyst implantation, which requires coordinated expression of maternal and embryonic genes. The fertilized egg undergoes dynamic genetic programming and divisions to reach the blastocyst stage. The pluripotent inner cell mass of the blastocyst will develop into the embryonic proper, while the trophectoderm and the primitive endoderm form extra-embryonic tissues during development [67]. Preimplantation embryonic development largely depends on maternal proteins and transcripts before zygotic genome activation (ZGA), which initiates the expression of genes that are needed for continued development of the embryos. ZGA occurs at the two-cell stage in the mouse [68].

Blastocyst implantation is a complex event that is controlled by both intrinsic embryonic programs and extrinsic cues including hormonal and uterine signals. Implantation in the mouse can be divided into three phases: apposition, attachment, and penetration. Following attachment, uterine stromal cells extensively proliferate and differentiate into decidual cells (i.e., decidualization) [69]. The roles of steroid hormones, cytokines, growth factors, integrins, and angiogenic factors have been explored, and more recently, a number of novel genes/pathways underlying implantation have been identified. Several elegant reviews are available on these topics [70–72]. The important roles of embryonic TGFβ superfamily signaling in embryo development have been reviewed [3]. This article will focus on the role of maternal TGFβ signaling in implantation and embryonic development.

TGFBs1-3 are founding members of the TGFβ superfamily. The majority of currently available studies are confined to the identification of tissue/cell-specific expression of TGFBs and *in vitro* analysis of the ligand function. In the uterus, the *in vivo* role of TGFβ signaling remains elusive, partially because of the redundancy of the ligands [73, 74] and the lack of appropriate animal models as a result of the embryonic lethality in mice lacking TGFβ ligands. TGFB1 is involved in preimplantation development and yolk sac vasculogenesis/hematopoiesis [75]. To allow the *Tgfb1* null mice survive to reproductive age, they were bred onto the severe combined immunodeficiency (*SCID*) background [76]. Although the uterus of *Tgfb1* mutant mice appears to be morphologically normal [76], embryos are arrested in the morula stage.

An *in vitro* model has been used to determine the effect of growth factors on preimplantation development, and the results showed that TGFB1 or epidermal growth factor (EGF) dramatically improves the inferior development of singly cultured embryos between eight-cell/morula and blastocyst stages. This study suggests that embryo and/or reproductive tract-derived growth factors are involved in the development of preimplantation embryos [77]. *In vitro* treatment of preimplantation stage embryos with TGFB1 increases total numbers of cells in expanded and hatching blastocysts [78]. Furthermore, TGFB1-promoted *in vitro* blastocyst outgrowth is blocked by an antibody directed to parathyroid hormone-related protein [79], which suggests the involvement of parathyroid hormone-related protein in mediating the effect of TGFB1 on blastocyst outgrowth. In addition, TGFB1 increases the *in vitro* expression of oncofetal fibronectin, an anchoring trophoblast marker, indicating a potential role of TGFβ in trophoblast adhesion during implantation [80]. TGFB1 also inhibits human trophoblast cell invasion, at least partially, by promoting the production of tissue inhibitor of metalloproteinases (TIMP) [81]. An elegant study showed that maternal TGFB1 can cross the placenta and rescue the developmental defects of *Tgfb1* null embryos, leading to perinatal survival of these mice [82]. As further evidence, both maternal and fetal TGFB1 may act to maintain pregnancy [83].

### TGFβ signaling and uterine diseases

#### Uterine fibroids

Leiomyoma, generally known as uterine fibroid, is a benign tumor arising from the myometrium (i.e., smooth muscle layers). Although leiomyoma is commonly benign, it could be the cause of fertility disorders and morbidity and mortality in women [84].

Increasing lines of evidence point to the involvement of TGFβ signaling in the development of leiomyoma. It has been shown that the expression of TGFBs and receptors is elevated in leiomyomata versus unaffected myometrium [85]. Among all the three TGFβ isoforms, TGFB3 seems to play a major role in leiomyoma development by promoting cell growth and fibrogenic process [86]. *Tgfb3* transcript and protein levels are elevated in human leiomyoma cells, compared with myometrial cells in two-dimensional (2D) and 3D cultures [87–90]. In a 3D culture system, a higher level of TGFB3 and SMAD2/3 activation is present in the leiomyoma cells versus myometrial cells [87, 89]. However, it does not support that connective tissue growth factor 2 (CCN2/CTGF) is a major mediator of TGFβ action in leiomyoma tissues [91].

Although a link between overexpression of TGFBs and leiomyoma has been recognized, the precise mechanisms of TGFβ signaling in leiomyoma are largely unknown. It has been demonstrated that TGFB1-stimulated expression of fibromodulin may contribute to the fibrotic properties of leiomyoma [92]. Moreover, treatment of myometrial cells with TGFB3 promotes the expression of ECM components such as collagen 1A1 (COL1A1), fibronectin 1 (FN1), and versican, but reduces the expression of those associated with ECM degradation [88, 93]. Thus, TGFβ signaling induces molecular changes that facilitate leiomyoma formation. Consistent with the enhanced TGFβ signaling in the etiology of leiomyoma, a number of substances or drugs, such as genistein [94], relaxin [95], halofuginone [96], asoprisnil [97], gonadotropin-releasing hormone-analogs (GnRH-a), and tibolone [98] may influence leiomyoma development via affecting TGFβ signaling. For the therapeutic purpose, an ideal drug is one that only targets TGFβ signaling in the leiomyoma cells but not normal myometrial cells. In this vein, asoprisnil, a steroidal 11β-benzaldoxime-substituted selective progesterone receptor modulator (SPRM), targets TGFB3 and TGFBR2 in leiomyoma cells but not normal myometrial cells [97], providing a potentially effective treatment option for leiomyoma. The high levels of leiomyoma-secreted TGFBs, in turn, may compromise uterine function of the patients. For example, by producing excessive amount of TGFB3, leiomyoma antagonizes decidualization mediated by BMP2 [99].

#### Preeclampsia

Preeclampsia often occurs in pregnant women after the 20^th^ week of gestation, characterized by hypertension and proteinuria. The causes of preeclampsia are complex and beyond the scope of this review. It has been shown that plasma TGFB1 [100–104] and TGFB2 [105] levels are elevated in patients with preeclampsia. Experimental evidence also suggests that failure to downregulate the expression of TGFB3 during early gestation may cause trophoblast hypo-invasion and preeclampsia [106]. Interestingly, the levels of soluble endoglin, a transmembrane TGFβ co-receptor, are elevated in sera of women with preeclampsia, which may be associated with vascular complications and hypertension in these patients [107, 108]. Based on these findings, TGFB proteins may serve as potential biomarkers for preeclampsia [105]. It is thus plausible that optimal TGFβ signaling activity is required to keep preeclampsia in check by maintaining normal trophoblast invasion during implantation and placentation. However, another study showed that TGFBs1-3 are not expressed in villous trophoblasts, and TGFB1 and TGFB3 are not expressed in the extravillous trophoblast either. The expression of TGFBs1-3 in the placenta is not altered in patients with preeclampsia [109]. Moreover, there are also reports indicating that concentrations of TGFB1 in serum are indistinguishable between patients with preeclampsia and normal controls [110–112]. In addition, the levels of activin A and inhibin A, but not inhibin B, are increased in patients with preeclampsia [113–116]. Thus, the role of TGFβ signaling in the pathophysiological events of preeclampsia awaits further elucidation.

#### Intrauterine growth restriction

Intrauterine growth restriction (IUGR), also called fetal growth restriction (FGR), refers to a complication of fetal growth during pregnancy. The estimated weight of the fetus with IUGR is often less than 90% of other fetuses at the same stage of pregnancy [117]. Circumstantial evidence indicates that TGFβ signaling is involved in the development of IUGR. Serum levels of TGFB1 in the IUGR fetus are lower [118]. TGFB2 is required for normal embryo growth, as supported by the fact that *Tgfb2* mutant fetuses weigh less than littermate controls [119]. Soluble endoglin levels are elevated in IUGR pregnancies [108], although it is debatable [120]. It has been shown that the higher expression of endoglin in IUGR pregnancies may be caused by placental hypoxia involving TGFB3 [121]. Mouse models for IUGR are valuable to study the mechanism of this pathological condition, which may have devastating effects on the pregnancy and newborns. Notably, *Nodal* knockout mice show diminished decidua basalis due to reduced proliferation and enhanced apoptosis as well as defects in placental development, resulting in IUGR and preterm fetal loss [122]. Conditional ablation of *Bmpr2* in the uterus causes defects in decidualization, trophoblast invasion, and vascularization, which are causes of IUGR in the pregnant females [46].

#### Endometrial hyperplasia

Endometrial hyperplasia is a pathological condition where endometrial cells undergo excessive proliferation [123]. Categories of endometrial hyperplasia include simple hyperplasia, simple atypical hyperplasia, complex hyperplasia, and complex atypical hyperplasia [124]. Endometrial hyperplasia is recognized as a premalignant lesion of endometrial carcinoma [125] and a potential cause of abnormal uterine bleeding and fertility disorders. The high prevalence of endometrial carcinoma is associated with atypical hyperplasia in women [126–128]. It has been reported that up to 29% of untreated complex atypical hyperplasia progresses to carcinoma [124]. Endometrial hyperplasia is generally caused by excessive or chronic estrogen stimulation that is unopposed by progesterone, as in patients with chronic anovulation and polycystic ovary syndrome. Although progestin treatment is commonly effective for this disease [129], approximately 30% of patients with complex hyperplasia are progestin resistant [130]. Genetic alterations including mutations of *Pten* tumor suppressor have been shown to be associated with endometrial hyperplasia [131, 132]. Elegant work has shown that inactivation of TGFβ signaling and loss of growth inhibition are associated with human endometrial carcinogenesis [133, 134]. The role of TGFβ signaling in endometrial cancer has been reviewed and will not be covered in this article [135]. Our recent study shows that loss of TGFBR1 in the mouse uterus using *Amhr2*-Cre enhances epithelial cell proliferation. The aberration culminates in endometrial hyperplasia. Further studies have uncovered potential TGFBR1-mediated paracrine signaling in the regulation of uterine epithelial cell proliferation, and provided genetic evidence supporting the role of uterine epithelial cell proliferation in the pathogenesis of endometrial hyperplasia [136]. Further elucidating the role and the underlying mechanisms of TGFβ signaling in the pathogenesis of endometrial hyperplasia and/or cancer will benefit the design of new therapies.

## Conclusions and future directions

A precisely controlled endogenous TGFβ signaling system is of critical importance for the development and function of female reproductive tract. Mouse genetics has proven to be a powerful tool to address many of the fundamental questions posed in the field of TGFβ and reproduction. Conditional knockout approaches have been utilized over the last two decades to decipher the reproductive function of TGFβ superfamily in female reproduction. These studies are at an exciting stage and are advancing at a rapid pace. The functional role of TGFβ signaling in the uterus is beginning to be unveiled. We anticipate that the genetic approach will continue to have large impacts and lead to new breakthroughs in this field. However, understanding how the hormonal, cellular, and molecular signals induce a specific biological response and functional outcome in the context of the uterine microenvironment *in vivo* represents a challenging task. It remains unclear how specific or integrated signals act on the chromatin to shape the epigenetic landscape in physiological and/or pathological conditions of the uterus. Therefore, the interaction between TGFβ signaling and other regulatory pathways (e.g., small RNA pathways) and potential epigenetic mechanisms underlying specific reproductive processes and/or diseases in the uterus need to be clarified. This knowledge will help to design new treatment options for uterine diseases and fertility disorders.
